# Supplementary material for: Identification of photoperiod-induced specific miRNAs in the adrenal glands of Sunite sheep (Ovis aries)
Source: Front Vet Sci. 2022 Jul 22;9:888207. doi: 10.3389/fvets.2022.888207 (PMC9354845; doi:10.3389/fvets.2022.888207)
Supplement: Supplementary file 1 [file Data_Sheet_1.ZIP › Supplementary file/miRDeep2 file.zip!/miRDeep2 file/Know_Structure/LP42A2/oar-mir-127.pdf]

miRBase precursor : oar-mir-127
Total read count : 116826
oar-miR-127 read count : 114510
remaining reads : 2316

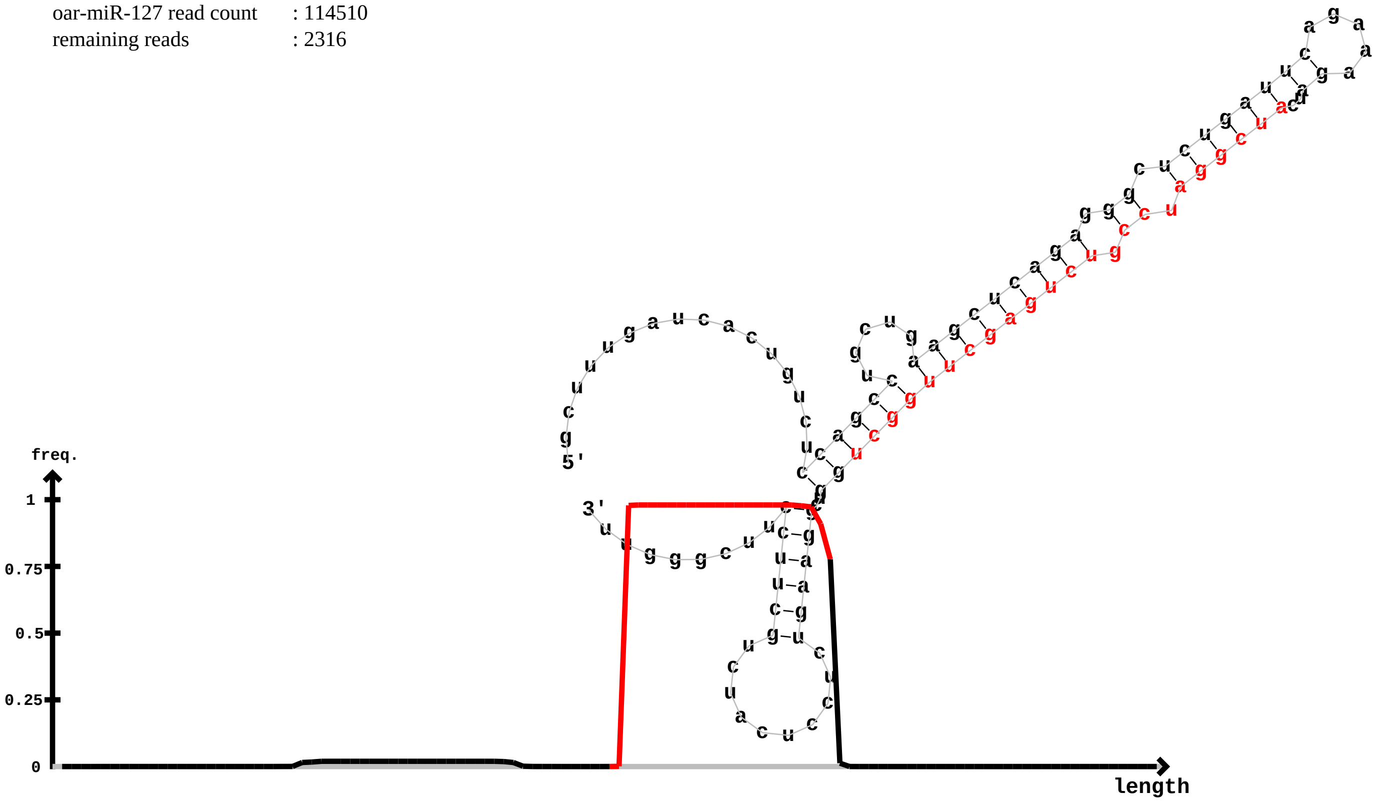


```
                                                            oar-miR-127
5'- gcuuugaucacugucuccagccugcugaagcucagagggcucugauucagaaagaucaucggauccgucugagcuuggcuggucggaagucuccucaucugcuuccuucggguu   -3'    exp
    ................((((((.....(((((((((.((.((((((((.....))..)))))).)).)))))))))))))))..((((((..........))))))........    reads      mm        sample
    ..uuugaucacugucuccagccug..........................................................................................    1          0         seq
    ...uugaucacugucuccagccug..........................................................................................    5          0         seq
    ....ugaucacugucuccagccug..........................................................................................    2          0         seq
    ......................ugcugaagcucagagggcucu.......................................................................    1          0         seq
    ......................ugcugaagcucagagggcucug......................................................................    7          0         seq
    ......................ugcugaagcucagagggcucuga.....................................................................    11         0         seq
    ......................ugcugaagcucagagggcucugau....................................................................    2          0         seq
    ......................ugcugaagcucagagggcucugauu...................................................................    2          0         seq
    .......................gcugaagcucagagggcucuga.....................................................................    6          0         seq
    .......................gcugaagcucagagggcucugau....................................................................    3          0         seq
    .......................gcugaagcucagagggcucugauu...................................................................    3          0         seq
    ........................cugaagcucagagggcuU........................................................................    1          1         seq
    ........................cugaagcucagagggcucC.......................................................................    1          1         seq
    ........................cugaagcucagagggcucug......................................................................    11         0         seq
    ........................cugaagcucagagggcucuga.....................................................................    92         0         seq
    ........................cugaagcucagagggcucugau....................................................................    390        0         seq
    ........................cugaagcucagagggcucugauu...................................................................    1230       0         seq
    ........................cugaagcucagagggcucugauuc..................................................................    17         0         seq
    ........................cugaagcucagagggcucugauucagaa..............................................................    2          0         seq
    .........................ugaagcucagagggcucugau....................................................................    14         0         seq
    .........................ugaagcucagagggcucugauu...................................................................    177        0         seq
    .........................ugaagcucagagggcucugauuc..................................................................    4          0         seq
    ..........................gaagcucagagggcucugau....................................................................    5          0         seq
    ..........................gaagcucagagggcucugauu...................................................................    197        0         seq
    ..........................gaagcucagagggcucugauuc..................................................................    108        0         seq
    ..........................gaagcucagagggcucugauuca.................................................................    8          0         seq
    ...........................aagcucagagggcucugauu...................................................................    8          0         seq
    ...........................aagcucagagggcucugauuca.................................................................    1          0         seq
    ...........................aagcucagagggcucugauucag................................................................    3          0         seq
    ...........................aagcucagagggcucugauucaga...............................................................    2          0         seq
    ...............................................cagaaagaucaucggauccgucugagcuu......................................    1          0         seq
    ...............................................cagaaagaucaucggauccgucugagcuuggc...................................    1          0         seq
    .......................................................ucaucggauccgucugagcuuggc...................................    1          0         seq
    .......................................................ucaucggauccgucugagcuuggcu..................................    1          0         seq
    ........................................................caucggauccgucugagcuugg....................................    1          0         seq
```

oar-miR-127

```
gcuuugaucacugucuccagccugcugaagcucagagggcucugauucagaaagaucaucggauccgucugagcuuggcuggucggaagucuccucaucugcuuccuucggguu
........................................................caucggauccgucugagcuuggcu..................................    3       0       seq
.........................................................aucggauccgucugagcuu......................................    1       0       seq
.........................................................aucggauccgucugagcuug.....................................    2       0       seq
.........................................................aucggauccgucugagcuugg....................................    18      0       seq
.........................................................aucggauccgucugagcuuggc...................................    59      0       seq
.........................................................aucggauccgucugagcuuggcu..................................    4       0       seq
..........................................................ucggauccgucugagcu.......................................    8       0       seq
..........................................................ucggauccgucugagcuu......................................    343     0       seq
..........................................................ucggauccgucugagcuug.....................................    488     0       seq
..........................................................ucggauccgucugagcuugg....................................    7410    0       seq
..........................................................ucggauccgucugagcuuggc...................................    15422   0       seq
..........................................................ucggauccgucugagcuuggcu..................................    89105   0       seq
..........................................................ucggauccgucugagcuuggcug.................................    1436    0       seq
..........................................................ucggauccgucugagcuuggcugg................................    13      0       seq
...........................................................cggauccgucugagcuug.....................................    1       0       seq
...........................................................cggauccgucugagcuugg....................................    15      0       seq
...........................................................cggauccgucugagcuuggc...................................    24      0       seq
...........................................................cggauccgucugagcuuggcu..................................    134     0       seq
...........................................................cggauccgucugagcuuggcug.................................    17      0       seq
...........................................................cggauccgucugagcuuggcugg................................    1       0       seq
............................................................ggauccgucugagcuuggcu..................................    2       0       seq
............................................................ggauccgucugagcuuggcugg................................    1       0       seq
```
